# Pharmacokinetics of Carprofen Administered Intravenously at Different Doses in Goats

**DOI:** 10.3390/vetsci12090852

**Published:** 2025-09-02

**Authors:** Orhan Corum, Halis Oguz, Mustafa Hitit, Duygu Durna Corum, Devran Coskun, Teslime Erdogan, Emre Bahcivan, Kamil Uney

**Affiliations:** 1 Department of Pharmacology and Toxicology, Faculty of Veterinary Medicine, University of Hatay Mustafa Kemal, Hatay 31060, Türkiye; 2 Department of Pharmacology and Toxicology, Faculty of Veterinary Medicine, University of Selcuk, Konya 42003, Türkiye; halisoguz@selcuk.edu.tr (H.O.); kuney@selcuk.edu.tr (K.U.); 3 College of Agriculture, Food and Natural Resources, Prairie View University, Prairie View, TX 77446, USA; 4 Department of Pharmacology and Toxicology, Faculty of Veterinary Medicine, University of Siirt, Siirt 56100, Türkiye; devran.coskun@siirt.edu.tr; 5 Ministry of National Education, Directorate of Lifelong Learning, Yenimahalle, Ankara 06560, Türkiye; 6 Department of Medical Pharmacology, Faculty of Medicine, Amasya University, Amasya 05100, Türkiye

**Keywords:** carprofen, pharmacokinetics, different doses, goat

## Abstract

This investigation focuses on understanding the pharmacokinetic behavior of intravenously administered carprofen at doses of 0.7, 1.4, and 4 mg/kg in goats. Eighteen animals were randomly assigned into three groups, with six goats per group, and blood samples were collected at 22 time points post-administration. Plasma concentrations were analyzed using a validated HPLC-UV method, and key pharmacokinetic parameters were derived using non-compartmental analysis. Results show that, at 0.7 mg/kg, carprofen exhibited the total clearance (Cl_T_) of 2.19 mL/h/kg, volume of distribution at steady state (V_dss_) of 126.56 mL/kg, area under the curve (AUC_0-last_) of 321.00 h*µg/mL, and elimination half-life (t_1/2ʎz_) of 44.32 h. When comparing doses, an increase in Cl_T_ was observed at 4 mg/kg, and the volume of distribution increased at both 1.4 and 4 mg/kg dosages. Additionally, a reduction in dose-normalized AUC_0-last_ was evident at the highest dose. Data showed that the effect of the drug may be prolonged as the dose increases. These differences between dose groups may be clinically insignificant after the single administration of all doses. Carprofen can be used at all three dose levels in goats; however, vigilance regarding the potential side effects and drug residues is essential, especially during repeated treatments.

## Simple Summary

This study investigates the pharmacokinetics of carprofen, an anti-inflammatory drug, when administered intravenously to goats at different doses. Key findings indicate that carprofen had a prolonged effect in goats, with a longer half-life and a slower clearance rate at all doses. As the dose increased, carprofen’s clearance accelerated, dose-normalized plasma concentrations decreased, and the volume of distribution expanded. However, despite the changes observed at higher doses, carprofen exhibited a longer duration of action at higher doses based on the reported therapeutic threshold. Therefore, the differences identified in this study are clinically negligible for the single administration of all doses. However, higher doses can lead to potential side effects and drug residues, especially with repeated treatments. This research provides valuable information for veterinarians considering the use of carprofen in goats. While higher doses may provide better efficacy, careful monitoring is essential. Further studies are needed to optimize dosing regimens and ensure the safe and effective use of carprofen in goat medications.

## 1. Introduction

Goats are small ruminants classified under the species *Capra hircus*, recognized as the first and most extensively domesticated livestock. The global goat population is estimated to be around 1 billion, predominantly located in the developing nations of Africa and Asia [1]. Goat breeding is common in Türkiye, accounting for 15% of the ruminant population [2]. Goats play an important role in animal meat and milk production, and their widespread breeding is due to their tolerance to high temperatures and prolonged water shortages, disease resistance, and ability to use degraded pasture areas [3].

Goats may have painful and inflammatory conditions as a result of castration, disbudding, injury, surgery, and bacterial infections [4,5]. Effective pain management is crucial for animal welfare and the production of high-quality products. α2-adrenergic agonists, opioids, and nonsteroidal anti-inflammatory drugs (NSAIDs) are used in the management of analgesia in small ruminants [6]. Carprofen, 6-chloro-alpha-methyl-9H-carbazole-2-acetic acid, is an NSAID with analgesic, antipyretic, and anti-inflammatory pharmacological effects and inhibits cyclooxygenase (COX) enzymes that synthesize prostaglandins from arachidonic acid [7]. NSAIDs having a COX-1/COX-2 selectivity ratio greater than 1 for inhibitory concentration (IC)_50_ are regarded as more effective in suppressing COX-2 [8], and carprofen has an inhibitory effect especially on the COX-2 enzyme in sheep (5.3–6.3) and dogs (1.75–5) [9,10]. Carprofen has a single chiral molecule, and the commercially available formulation is a 50:50 racemic combination of the S (+) and R (−) enantiomers [11]. It is used in the treatment of respiratory disease, mastitis, osteoarthritis, musculoskeletal pain, and trauma in cattle, horses, and canines [11].

Carprofen is not licensed for use in goats and is thus used extra-label for pain and inflammatory conditions. Carprofen has been used successfully in goats for castration [12], postoperative pain after claw amputation [6], and musculoskeletal pain [13]. The pharmacological properties of carprofen, such as its longer half-life than other NSAIDs used in veterinary medicine in horses, cattle [14], and sheep [15] and its lower risk of gastric irritation in rats than aspirin and indomethacin [16], make its use in goats advantageous. Although carprofen has no approved dose in goats, it is approved for use in cattle at 1.4 mg/kg and horses at 0.7 mg/kg intravenously or subcutaneously [14]. Therefore, carprofen is administered to goats at different doses (1.4–4 mg/kg), with a recommendation for repetition after 48–72 h if considered required [5,6,17]. However, dose-related alterations in pharmacokinetic parameters, including elimination half-life, volume of distribution, and clearance, have been documented in sheep [18,19]. This change in pharmacokinetics may alter the therapeutic efficacy and adverse effects of the drug. Furthermore, the pharmacokinetics of carprofen exhibited considerable variation between animal species [15,18,19,20,21,22,23]. Therefore, it is particularly important to reveal species- and dose-associated pharmacokinetic changes. The pharmacokinetics of carprofen have only been determined in castrated goats [12]; further studies are not available. This study hypothesizes that higher doses of carprofen could change how the drug is distributed, metabolized, and excreted in goats, and understanding these changes could help veterinarians to determine more effective and safe dosages. This study aims to investigate the pharmacokinetic changes of intravenously administered carprofen at different doses (0.7, 1.4, and 4 mg/kg) in goats.

## 2. Materials and Methods

### 2.1. Chemicals

Carprofen, with a standard purity of 97.0%, was obtained from Sigma-Aldrich (St. Louis, MO, USA). High-performance liquid chromatography-grade methanol was procured from VWR International (Fontenay-sous-Bois, France). Sodium acetate, acetic acid, perchloric acid, and n-butyl acetate were acquired from Merck (Darmstadt, Germany). The commercial formulation of carprofen (Rimadyl 50 mg/mL, Injection Solution, Zoetis Deutschland GmbH, Berlin, Germany) was utilized for medication administration in goats.

### 2.2. Animals

Eighteen healthy female goats (2.20 ± 0.17 years old), weighing 30–40 kg (average, 33.89 ± 2.68 kg), were used in this study. Goats that were judged to be healthy based on anamnesis and clinical examination were included in the study. Furthermore, goats were not administered for any medicine for a duration of two months before the trial. Ear tags and numbered collars were used to facilitate identification of the goats. The goats were taken to the enclosures chosen for the study and acclimatized to the surroundings during a two-week period. Goats were fed commercial feed suitable for their age and weight twice daily, at 8:00 a.m. and 8:00 p.m. Alfalfa hay and water were supplied ad libitum. The Local Ethics Committee for Animal Research Studies at Siirt University approved the study protocol (2021/05-44).

### 2.3. Experimental Design

For drug administration and blood collection, intravenous catheters (22 G, 0.9 × 25 mm) were inserted into the right and left jugular veins of goats, respectively. The research with goats was performed utilizing a parallel pharmacokinetic design. Goats were randomly assigned to three dose groups, each including six animals. Carprofen was administered intravenously at doses of 0.7 mg/kg for the first group, 1.4 mg/kg for the second group, and 4 mg/kg for the third group. Blood samples (2 mL) were collected in lithium heparin tubes at 0, 5, 10, 15, 30, and 45 min and 1, 2, 4, 6, 8, 10, 12, 24, 48, 72, 96, 144, 192, 240, 288, and 360 h. Blood samples were collected via catheter for the first 12 h and by venipuncture from both jugular veins at other sampling times. The blood was centrifuged at 4000 rpm for 10 min within one h, and the obtained plasma samples were stored at −80 °C until analysis.

### 2.4. HPLC Conditions

The HPLC system (Shimadzu, Tokyo, Japan) included a pump (model LC-20AT), a degasser (model DGU-20A), an auto-sampler (model SIL 20A), a column oven (model CTO-10A), and a UV detector (model SPD-20A). The analysis was conducted with LC Solution software (Version 1.25 SP5, Shimadzu Corp.). The injection loop volume was set at 20 μL. A Gemini^TM^ C18 column (4.6 × 250 mm; 5 μm) was used for the chromatographic separation of carprofen at a temperature of 40 °C. The mobile phase consisted of methanol (70%) and 0.2% perchloric acid (50 μL) in water (30%) with a flow rate of 1 mL/min. The wavelength for quantification was established at 254 nm.

### 2.5. Validation of the Analytical Method

Carprofen stock solution (1 mg/mL) was prepared in methanol. Working standards (0.02–80 μg/mL) were created by diluting the stock solution with ultrapure water. Calibration standards (0.02–80 μg/mL) and quality control samples (0.1, 4, and 40 μg/mL) were created by mixing the stock solution or working standard with blank goat plasma. The quantitative HPLC method was validated for goat plasma sample for linearity, recovery, accuracy, and precision, in accordance with EMA guidelines [24]. The method’s linearity was assessed by a calibration curve ranging 0.02–80 μg/mL of carprofen. Quality control samples were examined in six replicates on five different days to determine recovery, accuracy, and precision. The recovery was calculated by comparing the quality control samples with the working standards. The precision was evaluated using the percentage coefficient of variation (CV), whilst accuracy was defined as bias.

### 2.6. Sample Extraction

The samples were prepared according to a previously published method [15,25]. Three hundred microliters of acetate buffer (1 M, pH: 2.8) was combined with two hundred microliters of plasma and vortexed for 45 s. Two milliliters of n-butyl acetate was added to the mixture, vortexed for 45 s, and subsequently centrifuged at 12,000× *g* for 15 min. The upper phase was transferred to a separate tube and evaporated at 40 °C under nitrogen. The residue was dissolved in 200 μL of mobile phase, and 20 μL of this solution was injected into the HPLC system.

### 2.7. Pharmacokinetic Analysis

The chromatogram data acquired from the analysis of each goat were documented in an Excel file. Subsequently, the concentrations were computed, and plasma concentration-time profiles were constructed. The pharmacokinetic parameters were determined by non-compartmental analysis using Phoenix WinNonlin software (6.1.0.173, Certara, Inc., USA). The parameters calculated included the area under the curve (AUC) using the linear log trapezoidal, total clearance (Cl_T_), apparent volume of distribution (V_darea_), volume of distribution at steady state (V_dss_), elimination half-life (t_1/2λz_), mean residence time (MRT), and AUC extrapolated from tlast to ∞ in % of the total AUC (AUC_extrap_ %). The carprofen concentration at the first sampling time (C_0.083 h_) was determined from the individual plasma concentration. The body extraction ratio (E_body_) of carprofen was calculated for each goat by dividing clearance by cardiac output. Cardiac output was ascertained using the application of the allometric equation 180 × body weight (in kg)^−0.19^ [26].

### 2.8. Statistical Analysis

The statistical analysis was conducted using SPSS 22.0 (IBM Corp., Armonk, NY, USA). A *p*-value below 0.05 was deemed statistically significant. The t_1/2λz_ and MRT_0-∞_ were presented as harmonic mean (range) and other pharmacokinetic data were displayed as arithmetic mean (range). Levene and Shapiro–Wilk tests were used to assess homogeneity of variance and normality of data distribution, respectively. To determine statistically significant differences in pharmacokinetic variables between the three dose groups, the one-way analysis of variance (ANOVA) and post hoc Tukey test were used.

## 3. Results

### 3.1. Safety

No side effects were observed after single IV doses of carprofen 0.7, 1.4, and 4 mg/kg. Throughout the acclimatization and experimental phase, the behavior, feed and water intake, and fecal production of all goats were normal.

### 3.2. Analytical Method Validation

The analytical method exhibited remarkable linearity (R^2^ = 0.996) over the concentration range of 0.02–80 μg/mL. The lower limit of quantification for carprofen was 0.02 μg/mL, with a coefficient of variation under 9% and a bias of ±8%. The mean recovery of carprofen in goat plasma was >93%. The intra-day coefficients of variation, inter-day coefficients of variation values, intra-day bias, and inter-day bias of carprofen were <7.71%, <7.01%, <2.98%, and <2.44%, respectively.

### 3.3. Pharmacokinetics

**Figure 1:**
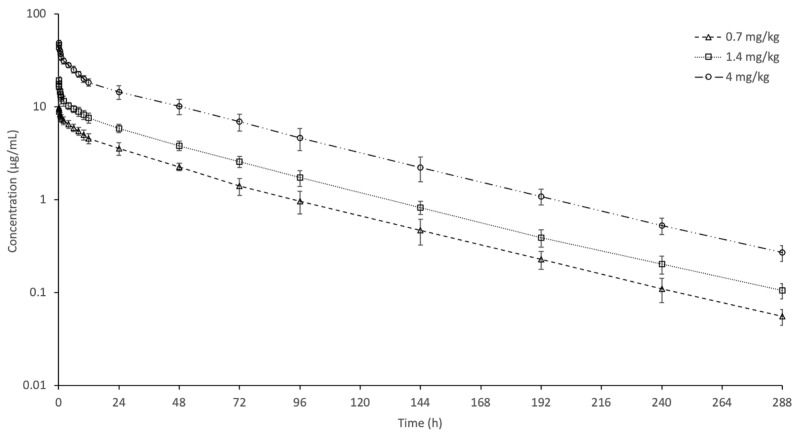
Semi-logarithmic plasma concentration–time curves of carprofen following single intravenous administration at doses of 0.7, 1.4, and 4 mg/kg in goats (n = 6, mean ± SD).

Figure 1 shows a semi-logarithmic plot of the mean plasma concentrations of carprofen in goats after IV injections of 0.7, 1.4, and 4 mg/kg. Carprofen was detected in goat plasma for up to 288 h in all dose groups. The C_0.083 h_ values were 9.75, 19.07, and 48.61 µg/mL for 0.7, 1.4, and 4 mg/kg, respectively. The concentrations at the last sampling time were 0.06, 0.11, and 0.27 µg/mL for 0.7, 1.4, and 4 mg/kg, respectively. Table 1 presents a summary of the pharmacokinetic parameters of carprofen. For the 0.7 mg/kg dose, t_1/2ʎz_, AUC_0-last_, V_dss_, and Cl_T_ were 44.32 h, 321.00 h*µg/mL, 126.56 mL/kg, and 2.19 mL/h/kg, respectively. The t_1/2ʎz_ of carprofen was very long at 44.32–45.83 h, and no difference was found between the dose groups. In the 4 mg/kg dose group, dose-normalized AUC decreased, while Cl_T_ and E_body_ increased compared to the 0.7 mg/kg group. The V_dss_ increased in the 1.4 and 4 mg/kg dose groups compared to the 0.7 mg/kg dose group. No dose-related change was observed in C_0.083 h_.

## 4. Discussion

Carprofen is utilized extra-label, as it is not approved for the treatment of pain and inflammation in goats. Since it is not approved in goats, the dosage regimens used are adapted from other animal species, resulting in differences in the dose in this case. Altering doses may affect the pharmacokinetics of the drugs, thereby influencing their therapeutic efficacy and adverse effects. Dose-associated changes in the pharmacokinetics of carprofen have been reported in sheep [18,19]. This study revealed the pharmacokinetics and dose-associated pharmacokinetic alterations of carprofen in goats for the first time. These results will contribute to the use of carprofen in goats in appropriate dosage regimens.

No local or systemic side effects were observed after administration of carprofen to goats at doses of 0.7, 1.4 and 4 mg/kg. The repeated use of carprofen in sheep (4–16 mg/kg, IV, every 24 h for 5 days), horse (1.4 mg/kg, oral, every 24 h for 14 days) and dog (9 mg/kg, oral, every 24 h for 14 days) caused no significant effect on hematological and biochemical parameters [20,21,27]. It has been stated that carprofen can be used intravenously in goats at doses of 1.4–4 mg/kg [5,6,17]. It is approved for intravenous use at a dose of 1.4 mg/kg in cattle and 0.7 mg/kg in horses [14]. Therefore, intravenous use of 0.7, 1.4 and 4 mg/kg doses was preferred in this study.

Interindividual variability in pharmacokinetic parameters is typically assessed using the coefficient of variation (CV%). Pharmacokinetic parameter variability is classified as “low” (CV% ≤ 10%), “moderate” (CV% around 25%), or “high” (CV% > 40%) [28]. In this study, the interindividual variability in pharmacokinetic parameters was moderate, with a CV of less than 20%. This suggests that carprofen pharmacokinetic parameters may demonstrate predictable individual values at each dose level. However, it is important to note that the goats selected for this study represent a more homogeneous population.

The t_1/2ʎz_ value of carprofen after IV injection in goats was approximately 45 h at all doses. The t_1/2ʎz_ of carprofen varies in animal species such as cattle (30.7–43.4 h) [29,30], sheep (26.1–45.57 h) [18,19], dogs (8.00–11.7 h) [20,31], horses (18.1–21.9 h) [21,32], cats (20.0 h) [33], and trout (30.66 h) [22]. These data indicate that carprofen has a long t_1/2ʎz_ duration in ruminants. No change was observed in t_1/2ʎz_ values depending on the carprofen dose in goats. However, the t_1/2ʎz_ was prolonged in sheep due to increased dose [18,19]. The disparity between goats and sheep may be attributed to variations in the parameters Cl_T_ and V_d_, of which t_1/2ʎz_ is a constituent [26].

V_darea_ and V_dss_ are the volume in pseudo-equilibrium and steady-state, respectively [34]. After IV injection at a dose of 0.7 mg/kg in goats, V_darea_ and V_dss_ were 140.41 and 126.56 mL/kg, respectively. The closeness of V_darea_ and V_dss_ values indicates that a minimum amount of carprofen was eliminated during the distribution phase. Similarly, carprofen showed small V_dss_ (116.49 mL/kg) in castrated goats when given at 4 mg/kg via the same route [12]. Carprofen administered intravenously at a dose of 0.7 mg/kg also exhibited small V_darea_ (95.5–250.0 mL/kg) and V_dss_ (92.7–220.0 mL/kg) in other animal species [19,32]. NSAIDs, which are acidic in nature and strongly protein-bound, are distributed unevenly throughout the body [35]. Carprofen is a weakly acidic substance with a propionic acid structure and a pKa value of 4.3. The plasma protein binding ratio in goats is unknown, but it exceeds 99% in dogs, horses, and cattle [14,31]. Therefore, the V_darea_ of carprofen is small, and its distribution to normal tissue is limited. The concentrations of liver and kidney in swine were 10 times lower than those in plasma [36]. However, it passes to the inflamed area at a higher ratio [32,37]. This situation is attributed to the physicochemical properties of the drug, the better passage of proteins to the inflammation area as microvascular integrity is disrupted, and the increase in blood flow in the area [35]. Compared to the 0.7 mg/kg, V_darea_ increased at the 4 mg/kg dose, whereas V_dss_ increased at both the 1.4 and 4 mg/kg doses. Previous research on sheep indicated that V_d_ increased with the dose [18,19]. Since carprofen is highly bound to plasma proteins, binding may reach saturation with increasing dose, which may lead to an increase in the amount of free drug in the blood and an increase in V_d_. However, in dogs, the binding of carprofen to plasma proteins was shown to be concentration-independent throughout the range of 1 to 40 μg/mL [31]. Moreover, blood albumin concentrations diminished (within 1–3 days) in horses following carprofen treatment [21]. Blood albumin levels were not assessed in this investigation; nevertheless, given that carprofen particularly binds to albumin, this reduction may have resulted in an increase in V_d_.

In the current study, the Cl_T_ of carprofen at a dose of 0.7 mg/kg was determined to be 2.19 mL/h/kg, consistent with findings in castrated goats (2.08 mL/h/kg) [12], sheep (1.98–2.5 mL/h/kg) [15,19], and calves (2.50 mL/h/kg) [29], but lower than those reported in dogs (17 mL/h/kg) [31], and swine (6.86 mL/h/kg) [36]. The E_body_ value for the same dose of carprofen in goats is 0.013, indicating a low extraction ratio (low E_body_ = 0.05) [26]. Similarly, sheep (E_body_ = 0.022) and calves (E_body_ = 0.031) have low E_body_ values [15]. This indicates that the elimination of carprofen is slow in ruminants. No data exists about metabolism and excretion of carprofen in goats. Carprofen is eliminated predominantly by biotransformation in humans, rats, and dogs, and is excreted mostly as ester glucuronide metabolites. The portion excreted unchanged in humans is less than 5% [38]. Its excretion varies among species; in humans it is excreted primarily through urine and in rats and dogs through bile. The ratios and pharmacokinetics of S (+) and R (−) enantiomers after the injection of racemic carprofen differed across animals [9,39]. The variation in the Cl_T_ level of carprofen between animal species may result from changes in metabolism and excretion. The Cl_T_ and E_body_ levels increased in the 4 mg/kg dosage group as compared to 0.7 mg/kg dose. No dose-related change in Cl_T_ was reported in the study conducted in sheep [18,19]. However, it was reported that Cl_T_ increased at the high dose after repeated administration to sheep at 1.4 and 4 mg/kg doses for 5 days [18]. In fact, the increase in Cl_T_ at the 4 mg/kg dose was a surprise for us. Prostaglandins are essential for renal hemostasis and renal perfusion, and NSAIDs exert their effects by inhibiting prostaglandins. This class of drugs, particularly when used in high or repeated doses, can reduce renal blood flow and, consequently, drug clearance due to prostaglandin inhibition [40,41]. However, it has been reported that carprofen has no effect on renal function in dogs [42,43]. The binding to plasma proteins affects the drug’s distribution and elimination, with Cl_T_ and V_dss_ being associated with the unbound fraction of the drug in plasma. The glomerular filtration ratio of drugs is inversely proportional to their plasma protein binding [44]. The increase in Cl_T_ value at the 4 mg/kg dose may be due to altered plasma protein binding.

Therapeutic research on the analgesic and anti-inflammatory properties of carprofen in goats is insufficient. Therapeutic plasma concentrations of carprofen are 7 μg/mL, 10–17 μg/mL, and >1.5 μg/mL for cats, dogs and horses, respectively [45,46,47]. While the plasma concentrations obtained at doses of 1.4 and 4 mg/kg were above the values reported in cats, dogs, and horses, the range specified for dogs was not reached in the 0.7 mg/kg dose group. When the plasma concentrations obtained in this study were evaluated, considering the therapeutic concentration (>1.5 μg/mL) reported for horses, it was determined that carprofen was effective goats for 48, 96, and 144 h after being injected with doses of 0.7, 1.4, and 4 mg/kg, respectively. These results showed that the duration of effect may increase depending on the dose. Carprofen is generally administered as a single dose, but it can be repeated after 48–72 h, if necessary, as indicated by guidelines for treating goats [17]. The study revealed that carprofen has a prolonged t_1/2ʎz_ of approximately 44.32 to 45.83 h in goats. Given this extended half-life, repeated administration may lead to accumulation in the body, increasing the risk of adverse effects and residue. Therefore, veterinary practitioners should exercise caution and closely monitor goats for any signs of toxicity or side effects when considering repeated dosing schedules. Regular assessment and adherence to withdrawal periods are also essential to ensure the safety and effectiveness of carprofen treatment in goats.

This study conducted in goats has some limitations. The fact that the plasma protein binding ratio, metabolism, and excretion routes of carprofen were not investigated in goats are among the limiting aspects. Carprofen is a racemic mixture, and the pharmacokinetics and activities of its enantiomers are different. The fact that the pharmacokinetics of enantiomers were not investigated is a shortcoming of this study. A further limitation of the study is the absence of evidence for the effects of carprofen on exudate pharmacokinetics and inflammatory indicators, including prostaglandin and thromboxane.

## 5. Conclusions

The findings show that, after single IV administrations of 0.7, 1.4, and 4 mg/kg doses, carprofen’s clearance accelerated with increasing dose, dose-normalized plasma concentrations decreased, and the volume of distribution expanded. However, despite the changes observed at higher doses, carprofen remained effective in goats for up to 48, 96, and 144 h following injections of 0.7, 1.4, and 4 mg/kg, respectively, based on the therapeutic threshold of >1.5 μg/mL reported for horses. Therefore, while the differences identified in this study are clinically negligible for the single administration of 0.7–4 mg/kg doses, it may be crucial to monitor for potential side effects and residue accumulation, particularly with repeated administration. Further research is warranted to explore the binding characteristics and enantiomer-specific pharmacokinetics of carprofen in goats to optimize therapeutic regimens and enhance safety profiles for veterinary applications.

## Figures and Tables

**Table 1:** Pharmacokinetic parameters of carprofen following single intravenous administration at doses of 0.7, 1.4, and 4 mg/kg in goats (n = 6).

Parameter	0.7 mg/kg	CV%	1.4 mg/kg	CV%	4 mg/kg	CV%
t_1/2ʎz_ (h)	44.32 (6.06)	4.90	45.40 (3.39)	2.96	45.83 (3.40)	2.63
AUC_0-last_ (h*µg/mL)	321.00 (116.22)	13.96	547.81 (192.43)	12.53	1424.79 (640.15) ^b^	16.96
AUC_0-∞_ (h*µg/mL)	324.55 (117.24)	13.96	554.75 (196.59)	12.64	1442.61 (649.08) ^b^	16.96
DN * AUC_0-last_ (h*µg/mL)	-	-	273.90 (96.22)	12.53	249.53 (112.11) ^b^	16.96
DN * AUC_0-∞_ (h*µg/mL)	-	-	277.37 (98.30)	12.64	252.65 (113.67) ^b^	16.96
AUC_extrap_ (%)	1.09 (0.58)	18.38	1.24 (0.32)	10.17	1.23 (0.31)	9.59
MRT_0-∞_ (h)	57.79 (9.98)	6.49	60.09 (5.84)	3.30	60.89 (7.98)	5.34
Cl_T_ (mL/h/kg)	2.19 (0.77)	13.66	2.56 (0.86)	12.00	2.84 (1.21) ^b^	16.33
V_darea_ (mL/kg)	140.41 (54.32)	14.52	167.09 (44.45)	9.55	187.95 (78.98) ^b^	17.27
V_dss_ (mL/kg)	126.56 (34.56)	11.39	153.24 (37.44) ^a^	9.10	172.10 (51.37) ^b^	12.14
C_0.08 h_ (µg/mL)	9.75 (2.62)	9.13	19.07 (5.73)	10.96	48.61 (10.01)	6.77
DN * C_0.08 h_ (µg/mL)	-	-	9.54 (2.86)	10.96	8.51 (1.75)	6.77
E_body_	0.013 (0.01)	18.42	0.016 (0.01)	17.77	0.018 (0.011) ^b^	14.50

DN *: Values normalized to a dose of 0.7 mg/kg. ^a^: There are significant differences between the 0.7 mg/kg and 1.4 mg/kg dose (*p* < 0.05). ^b^: There are significant differences between the 0.7 mg/kg and 4 mg/kg dose (*p* < 0.05). AUC; area under the concentration-versus time curve, AUC_extrap_ %; area under the plasma concentration–time curve extrapolated from t_last_ to ∞ in % of the total AUC, Cl_T_; total body clearance, C_0.08 h_; plasma concentration at 0.08 h, CV; coefficient of variation, E_body_; body extraction ratio, MRT_0-∞_; mean residence time, t_1/2λz_; terminal elimination half-life, V_darea_; apparent volume of distribution; V_dss_; volume of distribution at steady state.

## Data Availability

The data presented in this study is available on request from the corresponding author.
